# Sensory and Motor Function, Pain, and Health Status in Children with Arthrogryposis and Myelomeningocele †

**DOI:** 10.3390/children11121480

**Published:** 2024-12-03

**Authors:** Åsa Bartonek, Marie Eriksson

**Affiliations:** Department of Women’s and Children’s Health, Division of Paediatric Neurology, Karolinska Institutet, Karolinska University Hospital, S-17176 Stockholm, Sweden; marie.eriksson@ki.se

**Keywords:** somatosensory, two-point discrimination, vibration, pressure injuries, orthotics

## Abstract

Background/Objectives: Proprioception and sensory disorders have been reported in children with arthrogryposis multiplex congenita (AMC) and myelomeningocele (MMC), but valid and reliable assessment tools are limited in accurately identifying the sensory aspects of motor disorders. This study aimed to investigate the somatosensory status in the feet and legs. An additional purpose of this study was to explore pain, skin irritations, and health status. Methods: Nineteen children with AMC, twenty-three with MMC, and twenty-two typically developing (TD) children (7–18 years old) were tested using a somatosensory test battery in ankle kinesthesia and in identifying four different types of floors. Results: In the AMC and MMC groups, the threshold to perceive the somatosensory stimuli was not achieved by all participants. MMC participants perceived somatosensory stimuli less than TD participants in all tests, with a higher level of the lesion and more affected ambulation. The MMC group identified one floor significantly less often than the TD group. The AMC group performed better than the MMC group in two-point discrimination, vibration sensation, and some light-touch pressure tests. There were no differences among the TD, AMC, and MMC groups in ankle kinesthesia. Pain was reported by four (21%) subjects in the AMC group and five (22%) in the MMC group, and skin irritations were reported by three (13%) participants in the MMC group. There was no difference among the TD, AMC, and MMC groups in health status as reported using the EQ-5D-Y visual analog scale. Conclusions: Although differences in sensory aspects were the most evident between the groups, assessments of activity and participation levels in the rehabilitation of children with disabilities are also recommended.

## 1. Introduction

In orthopedic care and child rehabilitation, patients with arthrogryposis multiplex congenita (AMC) and myelomeningocele (MMC) are frequently observed. It is known that sensory and proprioception disorders can be linked to AMC and MMC. However, valid and reliable assessment tools for accurately identifying sensory deficits in children with neurological disorders are limited, and although therapists understand the importance of somatosensation, they are not confident with current somatosensory measures [1].

The term arthrogryposis is used to describe a heterogeneous group of affected individuals who are recognized as having multiple congenital contractures in the newborn period. All forms of arthrogryposis are associated with decreased fetal movement, indicating a direct relationship between the early onset of fetal akinesia and the severity of contractures. The most common type is amyoplasia, presenting with characteristic extended elbows and with a frequency of 1 in 10,000 [2]. The contractures are usually non-progressive and improve over time with early physiotherapy and appropriate orthopedic treatment. Treatments for AMC include nonsurgical approaches, such as splinting for upper extremity contractures, and surgical approaches for lower extremity contractures [2,3]. As reported in Canada, the prevalence during 1997–2007 for multiple congenital contractures was 1/4300 [4]. Researchers have proposed a new classification to specify diagnostic categories, such as limbs with and without noncentral nervous system anomalies. Since several neuromuscular disorders may include AMC and CNS/brain involvement, a careful genetic and neurological clinical evaluation has been emphasized when making a specific diagnosis [5]. In the western region of Sweden, all children with multiple congenital contractures born between 1979 and 1994 (n = 68) were identified, comprising 1 in 5100 live births, with cases including cerebral and spinal involvement, neuromuscular or connective tissue involvement, and those due to mechanical restriction within the uterus [6]. With AMC, several conditions with multiple congenital contractures are present, requiring careful evaluations to make a specific diagnosis. During neurologic examinations, a test is therefore recommended to determine if the sensation is intact [2]. Among the heterogeneous group of AMC cases, normal sensation has been reported in amyoplasia [7,8]. In the distal forms of AMC, the PIEZO2 gene has been reported to play a major role in light-touch mechanosensation and has been identified as the principal mechanotransduction channel for proprioception [9]. Mutations in PIEZO2 may also indicate a distinct clinical phenotype, presenting with sensory ataxia, a proprioception defect with dorsal column involvement, together with arthrogryposis [10].

MMC is characterized by the failure of the spinal neural tube to close during embryonic development and occurs in approximately 1 per 1000 births worldwide. It is one of the most common congenital malformations, with the spinal cord being open dorsally, forming a placode on the back of the newborn baby. This malformation often affects urinary and fecal functions, and it may be associated with hydrocephalus requiring shunting and with neurological deficits below the level of the lesion, thus resulting in lower-limb weakness or paralysis [11]. The muscle pareses that are present at various neurological levels cause instability, leading to difficulties in maintaining a stable upright position. For example, children who present with sufficient knee extensor muscle strength at the mid-lumbar level may theoretically achieve independent standing, while the calf muscles may already be paretic at a low lumbar level, causing instability in the ankle joint, thus impeding the functional alignment of joints and body segments [12]. In addition to flaccid paresis, additional neurological deficiencies, such as the tethering of the spinal cord [11], may be present, resulting in spasticity that, in turn, may deteriorate mobility [13]. Therefore, it should be taken into account that children with similar neurological lesion levels cannot be expected to achieve an ambulatory function equivalent to that of their peers with flaccid pareses [12,14]. Individuals with MMC exhibit neurological deficits involving sensory functions, with a lack of sensation enhancing the risk of pressure sores [11]. In the early 1970s, carrying out sensory testing on newborn children with MMC was suggested in order to determine the lowest level of normal sensation, with motor testing performed next [15]. In that study, sensory loss in the trunk and lower limbs was found to usually follow motor loss within one or two segments, whereas in most patients with sacral agenesis, the sensation was intact caudally for several segments beyond the motor level [16]. In an attempt to evaluate sensation in children with severe tactile deficits using both electrical stimulation and traditional neurological examination, similar results were achieved for 45% of subjects, while electrical stimulation frequently detected sensation at sites that were classified as anesthetic upon neurological examination [17].

Even though the diagnoses are different, both AMC and MMC groups often have difficulty in achieving an appropriate posture in the weight-bearing position. In both groups of MMC and AMC, utmost mobility function is achieved through external support by orthotics to counteract unstable joints caused by muscle weakness and orthopedic deformities. In the AMC group, knee extension and ankle-joint-stabilizing orthoses are common, whereas some children only require foot orthotics [18]. In the MMC group, orthoses may range from foot orthoses to orthoses that enclose the pelvis and trunk, depending on the neurological level and possible additional neurological disturbances of each patient [19].

In all everyday activities, we depend on signals originating from our moving bodies concerning the position and movement of the limbs and trunk via sensations arising in proprioceptors located in the skin, muscles, and joints; thus, to move towards a target, ongoing visual and proprioceptive feedback is necessary [20]. When examining the knee-joint-position sensing in children with AMC and MMC in a non-weight bearing position, no differences, compared to that in typically developing (TD) children, either with respect to age or the ambulatory level, were found [21]. From this study, researchers speculated that muscle activation of the innervated knee extensors contributed sufficiently to the ability to sense the knee position, considering the major role of kinesthetic sensors in muscle spindles [20]. It has also been considered that, despite the extensive loss of skin and joint sensation in the legs of patients with partial or total sections of their dorsal columns, the senses of position and movement are preserved, allowing kinesthetic information to pass through an intact remaining central projection pathway for muscle afferents [20]. In these cases, there is also a relative failure of proprioceptive postural control associated with muscle weakness, indicating a functional link between contractile and sensory muscular processes [22].

Numerous studies have been carried out to investigate motor function in children with various disabilities. Given the importance of identifying sensory loss, there is currently a lack of knowledge in the literature. In children with cerebral palsy (CP), which is a central nervous impairment that occurs in the developing brain, somatosensory deficits in the foot and ankle have been identified [23,24]. In the case of children with AMC, even if recommended, there is no known valid instrument to assess sensation. Even for children with MMC, who have sensory paresis by definition, there is no available validated assessment tool. This highlights a research gap. The main aim of this study was to investigate the somatosensory status in the feet and legs of children with MMC and AMC. The hypothesis was that these children would perceive somatosensory stimuli less than TD children. The secondary purpose of this study was to explore pain and health statuses among the children.

## 2. Materials and Methods

### 2.1. Participants

Of a total of 57 children with AMC and MMC who were contacted between February 2023 and May 2024, 42 children (17 females and 25 males, with a mean age of 12 (7–18) years) agreed to take part in this cross-sectional study. Of these, 19 participants had been diagnosed with AMC and 23 with MMC. Participants were required to meet the following criteria: knee extension muscle strength grade 4 or greater [25], no orthopedic surgery on the feet and ankles in the last year, and the ability to understand verbal instructions necessary to accomplish the test. A total of 22 individuals with typical development (TD) (15 females and 7 males, with a mean age of 12 (7–18) years) constituted a control group. Participants with motor disabilities were recruited at Karolinska University Hospital and at a prosthetic and orthotic clinic in Stockholm, Sweden. The TD children were recruited through advertisements at the hospital and the relatives of patients and colleagues. Ethical approval for the study was obtained from the Regional Ethical Review Board in Lund, Sweden (Dnr 2022-03064-01), and written informed consent was obtained from all participants.

### 2.2. Motor Function

Children with AMC were designated to a subgroup based on joint stabilization during gait; these subgroups were AMC1, requiring orthotic support above the knee; AMC2, requiring support of the ankle and foot; or AMC3, requiring mainly shoe insoles [18]. In the MMC group, motor function was defined using a muscle function class (MFC) [12] based on muscle strength in the lower limb muscles from the sacral (MFC I), low lumbar (MFC II), and mid-lumbar (MFC III) to high lumbar level (MFC IV). Ambulatory function was categorized as being either within the community (Ca), within the household (Ha), non-functional (N-f), or non-ambulation (N-a) [26,27], and the use of orthoses for gait was documented.

The passive joint range of motion in the ankle joint was measured with a goniometer.

Subgroups of AMC and MMC, the presence of shunted hydrocephalus in MMC, and orthopedic surgery performed on the legs were documented based on the medical chart.

### 2.3. Somatosensory Function

**Figure 1:**
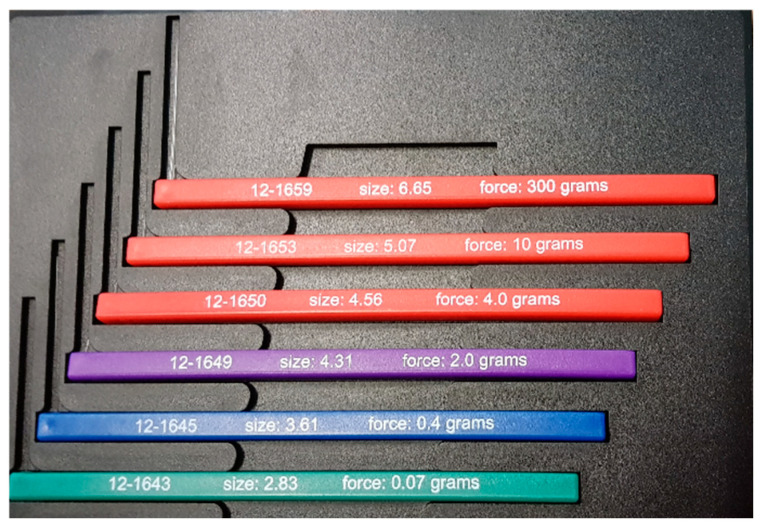
Six-item monofilaments of different diameters that measure the cutaneous sensory perception threshold.

Somatosensory tests were performed with the participants lying supine on an examination table, and kinesthesia tests were performed on the ankle when participants were sitting; the participants’ vision was covered during both tests. Light touch pressure on the plantar surface of the heel and at the first and fifth metatarsal head of the forefoot was assessed using a six-item monofilaments kit that measures the cutaneous sensory perception threshold (Baseline^®^, White Plains, NY, USA) [24] (Figure 1).

Each monofilament is of a different diameter and buckles when a specific force is applied to the skin. The test is performed by touching the skin with a monofilament in a perpendicular orientation three times in each area, in random order, and in variable time intervals. The light-touch-pressure threshold was recorded as the lowest monofilament value, at which the participant was able to correctly identify the stimulus twice for each location and limb. Light touch was also conducted on the mid-thigh, mid-shin, and mid-dorsum of the foot [23] with the monofilaments described above. 

Two-point discrimination was assessed on the plantar side of the forefoot and heel, and the procedure was performed randomly by using an aesthesiometer that evaluates cutaneous sensitivity and touch threshold discrimination (Baseline^®^, White Plains, NY, USA) [24]. The procedure begins by testing the largest distance (50 mm) to ensure the subject’s ability to detect the two different stimuli. If the subject correctly identifies this, the test will proceed with the smallest tested distance (10 mm); then, the distance is increased by 5 mm increments up to 45 mm until the threshold is reached. Three trials per condition (two-point stimuli, one-point stimulus, sham-trial) and location were performed in a randomized order, and the child was asked to report the number of perceived stimuli from the aesthesiometer (i.e., two, one, or none).

Vibration sensation was evaluated by using a 128 Hz tuning fork over the first metatarsal head and medial malleolus [24]. The examiner struck the tuning fork on a rubber-padded surface located next to the participant’s feet to elicit strong movement in the prongs. Then, the tuning fork was positioned perpendicular to the skin on the predefined bony prominences. The duration of the vibration stimulus was recorded by a chronometer, starting from the activation time of the tuning fork until the participant reported that they could not feel it. The average time, in seconds, of the three trials for each site was used for the analysis.

Joint kinesthesia is the ability to sense motion in a joint. With the ankle joint in a neutral position [24], the tested foot was grasped by the examiner on its lateral and medial edges, and the ankle joint was then passively moved towards plantarflexion or dorsiflexion in random order. Participants were asked to instantly report the direction of the displacement as “up” or “down”. Performance accuracy was determined as the number of correct responses out of 10 trials (5 in each direction) and was converted to a percent value. All tests were performed bilaterally.

### 2.4. Floor Sensation Test

**Figure 2:**
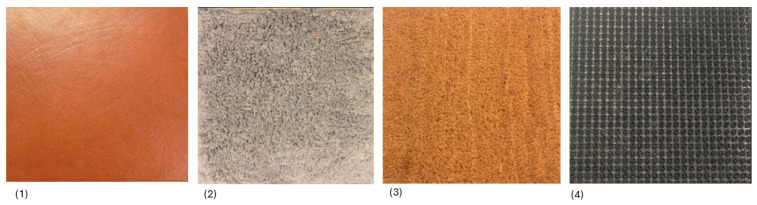
Floors of 30 cm × 30 cm of the following materials: (**1**) ceramic tile floor (“stone floor”), (**2**) textile (“soft bath carpet”), (**3**) coconut husk (hall mat indoor”), (**4**) plastic turf (“entrance mat outdoor”).

The participants’ ability to sense various floor textures was examined as follows: participants sat barefoot on a stool when sensing four different floors, with both feet, in randomized order, without vision, and with earplugs in order to reduce auditory help in identifying the “floors”. The floors, each mounted on a wooden plate, were 30 cm × 30 cm comprising the following materials: (1) ceramic tile floor (“stone floor”), (2) textile (“soft bath carpet”), (3) coconut husk (hall mat indoor”), (4) plastic turf (“entrance mat outdoor”) (Figure 2).

The mats were tested in randomized order 5 times each, i.e., 20 times in total. The child was initially allowed to feel the materials with both of their hands and feet and was provided pictures to point to for both the verbal and non-verbal identification of the floors. Three or more approved attempts for each floor were accepted as a pass.

### 2.5. Pain Assessment

The participant was asked whether pain was perceived in the lower limbs or back. If pain was present on examination day, a self-report measure, the Faces Pain Scale [28], was offered to the child for them to score the intensity of their pain; this scale has been recommended for use for children aged four or five onward.

Participants were also asked if they had pressure sores or skin irritations on their feet and legs.

### 2.6. Health Status Questionaire

The children were asked to fill in a questionnaire with five questions regarding their perceived current health status using EQ-5D-Y [29], which is a generic questionnaire for children 8 years or older. EQ-5D-Y contains five dimensions: mobility (walking about), looking after myself, performing regular activities, experiencing pain or discomfort, and feeling worried, sad, or unhappy. Each dimension contains three levels of severity: “no problems”, “some problems,” and “a lot of problems”. The children were asked to rate their overall health, as perceived on the examination day, using EQ-5D-Y visual analog scale (VAS), on a scale ranging from 0 (“worst imaginable health”) to 100 (“best imaginable health”) [29].

### 2.7. Statistics

Descriptive data are presented as the mean and SD, median, and minimum/maximum (min/max) values. The Kruskal–Wallis test with the post hoc Mann–Whitney *U* test was used to analyze answers from somatosensory tests, floor sensation, and VAS health status scores with respect to the TD, AMC, and MMC groups. The Mann–Whitney *U* test was used to identify the number of sensed vs. not-sensed stimuli among the TD, AMC, and MMC groups. A chi-square test was used to compare somatosensory tests and floor sensation between diagnosis subgroups and ambulation groups of participants with AMC and MMC. Statistical analyses were carried out using SPSS version 28.0. The significance level was set at *p* < 0.05.

## 3. Results

There were no differences in age, sex, ambulation, weight, height, or BMI between the groups of TD children, on the one hand, and children with AMC and MMC (Table 1). Sub-diagnoses, motor function, functional ambulation, and orthosis use in AMC and MMC groups are shown in Table 1.

A total of 11 of the 23 participants with MMC had shunted hydrocephalus (*p* = 0.835). Orthopedic surgery procedures on the legs were performed for 17/19 (89.5 %) children with AMC and for 11/23 (47.8%) children with MMC (*p* = 0.005).

### 3.1. Somatosensory Tests Not Perceived

Somatosensory stimuli were not perceived by participants with AMC in either the right or left leg, on the heel in 1/19 (*p* < 0.001), and on mid-thigh in 1/19 (*p* < 0.001). Vibration sensation was not perceived in either the right or left leg on the first metatarsal head in 8/19 children (*p* = 0.491) and was not perceived in the medial malleolus in 12/19 (*p* = 0.251). In three participants, a lack of attention did not permit vibration tests due to the participants’ young age; one of these participants was a TD child.

**Figure 3:**
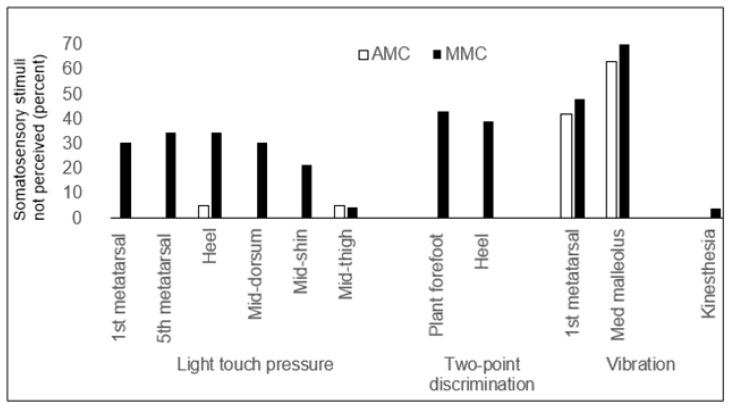
Somatosensory stimuli not perceived, shown as a percentage, in children with arthrogryposis (AMC) and myelomeningocele (MMC) in light touch pressure at the first metatarsal head, fifth metatarsal, heel, mid-dorsum, mid-shin, and mid-thigh; in two-point discrimination at plantar forefoot and heel; in vibration sensation at first metatarsal head and medial malleolus; and in ankle kinesthesia.

Somatosensory stimuli were not perceived by participants with MMC in either the right or left leg, on the first metatarsal head in 7/23 children (*p* = 0.061), on the fifth metatarsal in 8/23 (*p* = 0.144), on the heel in 8/23 (*p* = 0.144), on the mid-dorsum in 7/23 (*p* = 0.061), on the mid-shin in 5/23 (*p* = 0.007), and on the mid-thigh in 1/23 children (*p* < 0.001). Two-point discrimination in MMC was not perceived in either the right or left leg on the plantar forefoot in 10/23 children (*p* = 0.532) and was not perceived on the heel in 9/23 (*p* = 0.297). Vibration sensation was not perceived by participants with MMC in either the right or left leg on the first metatarsal head in 11/23 (*p* = 0.835) and on the medial malleolus in 16/23 (*p* = 0.061). One child with MMC did not perceive kinesthesia in the ankle joint. Figure 3 shows the somatosensory stimuli not perceived in the AMC and MMC groups, expressed as a percentage.

### 3.2. Somatosensory Tests Between Groups

The first and fifth metatarsals were perceived unilaterally by one participant each; the heel was perceived unilaterally by five, the mid-dorsum by six, and the mid-shin and mid-thigh by three participants. Two-point discrimination on the forefoot and on the heel were each perceived unilaterally by one participant. Two participants perceived one and two of three vibration trials, respectively, and one participant perceived kinesthesia in only one of the ankle joints. In those who perceived sensation in both legs, there were no differences between the right and left sides, and the sides were averaged. Table 2 shows light touch pressure, two-point discrimination, vibration sensation, and joint kinesthesia for groups of TD, AMC, and MMC patients.

**Figure 4:**
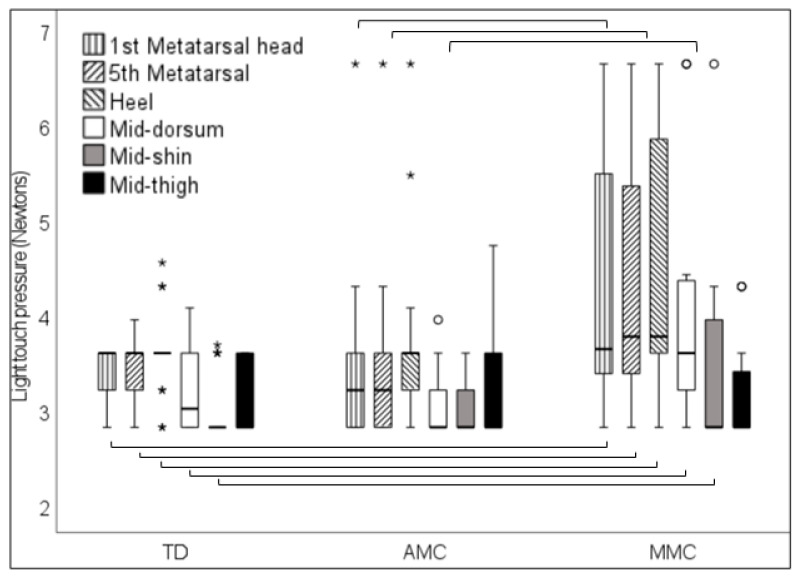
Light touch pressure in Newtons at the first metatarsal head, fifth metatarsal, heel, mid-dorsum, mid-shin, and mid-thigh in typically developing children (TD) and in children with arthrogryposis (AMC) and myelomeningocele (MMC). Parentheses indicate significant differences between groups. “○” = outlier, “*” = extreme outlier.

Higher thresholds during the testing of light touch pressure were found in MMC participants than in TD participants at the first metatarsal head (*p* = 0.005), fifth metatarsal (*p* = 0.017), heel (*p* = 0.011), mid-dorsum (*p* = 0.004), and mid-shin (*p* = 0.039) and higher thresholds in TD participants than in AMC participants were found at the first metatarsal head (*p* = 0.026), fifth metatarsal (*p* = 0.044), and mid-dorsum (*p* = 0.008) (Figure 4).

**Figure 5:**
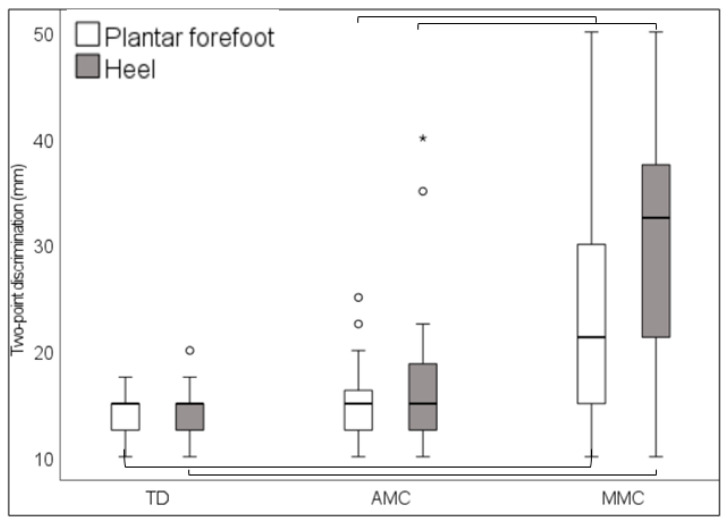
Two-point discrimination, in millimeters, on the forefoot and on the heel in typically developing children (TD) and in children with arthrogryposis (AMC) and myelomeningocele (MMC). Parentheses indicate significant differences between groups. “○” = outlier, “*” = extreme outlier.

According to two-point discrimination, larger distances were required for MMC participants than for TD subjects on the forefoot and on the heel (*p* = 0.005 and *p* < 0.001, respectively), and larger distances were required for MMC participants than for AMC participants (*p* = 0.006 and *p* = 0.003), respectively (Figure 5).

**Figure 6:**
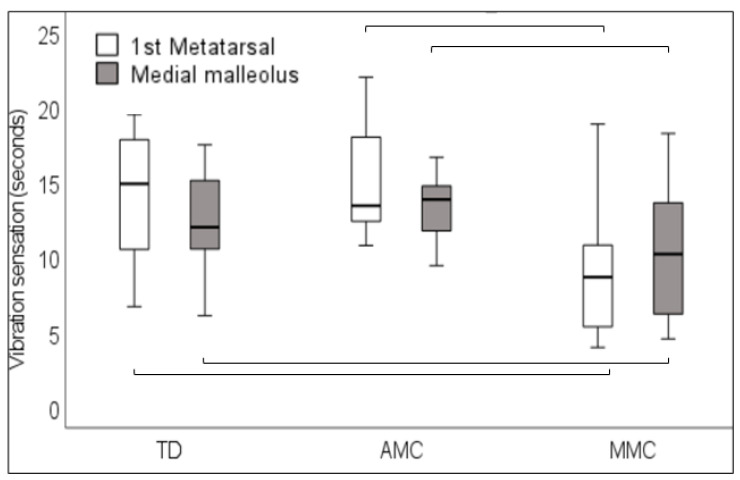
Vibration sensation, in seconds, on the first metatarsal head and medial malleolus in typically developing children (TD) and in children with arthrogryposis (AMC) and myelomeningocele (MMC). Parentheses indicate significant differences between groups.

The vibration time on the first metatarsal head and on the medial malleolus was perceived to be shorter by MMC participants than by TD participants (*p* < 0.001 and *p* = 0.026), respectively, and was perceived to be shorter by MMC participants than by AMC patients (*p* < 0.001 and 0.033, respectively) (Figure 6).

Kinesthesia in the ankle joint was performed for all participants, with 5 degrees or more in the ankle’s passive range of motion (ROM) in both ankles (n = 61) and in one ankle (n = 2). In one participant with AMC, five degrees of ankle ROM was not achieved, and one participant with MMC did not perceive any ankle joint movement. MMC participants had the lowest number of correct responses, but this number was not significantly lower (Table 2).

### 3.3. Floor Sensation

The floor sensation test was performed on all participants. Each floor was sensed in ≥3/5 trials by TD children.

In AMC participants, ≥3/5 trials were identified for Floor1 by 18/19 (*p* < 0.001) children, for Floor2 by all 19 (ns), for Floor3 by 16/19 (*p* = 0.003), and for Floor4 by 18/19 (*p* < 0.001).

In MMC participants ≥ 3 trials were identified for Floor1 by 16/23 (*p* = 0.061) children, Floor2 by 17/23 (*p* = 0.022) children, Floor3 by 10/23 (*p* = 0.532), and Floor4 by 19/23 (*p* = 0.002) children.

MMC participants identified ≥ 3/5 trials for Floor3 significantly less than TD children (10 vs. 22, *p* = 0.002).

In AMC, none of the floors was more identified than the other floors (*p* = 0.096). Floor3 was identified less frequently by MMC participants than Floor1 (*p* = 0.034), Floor2 (*p* = 0.008), and Floor4 (*p* = 0.003).

### 3.4. Subgroups

There were no differences in somatosensory tests and floor sensation in motor function among AMC1, AMC2, and AMC3, in functional ambulation between the Ca group and the Ha/N-f/N-a group, nor between the AMC sub-diagnosis amyoplasia group and patients with other forms of AMC.

In motor functions, both the MFC I/MFC II vs. the MFC III/MFC IV group and in functional ambulation, the Ca group vs. the Ha /N-f /N-a group passed more ankle kinesthetic trials and sensed Floor1 (*p* = 0.019), Floor2 (*p* = 0.048), and Floor3 (*p* = 0.002) more frequently.

### 3.5. Pain and Pressure Sores on Examination Day

Pain was reported by 4/19 participants with AMC and by 5/23 with MMC (*p* = 0.957). Skin irritations were reported by 3/23 participants with MMC; these included irritations on the back, on the first toe, and on the fifth toe.

### 3.6. Health Status

The generic health status questionnaire EQ-5D-Y was answered by 57 children with a mean age of 12.9 (7.3–17.9) years (Table 3).

## 4. Discussion

While there are commonly used tools for motor function and ambulation in children with motor disabilities, valid and reliable assessment tools for sensation are limited. Although therapists understand the importance of somatosensation, they are not confident with current somatosensory measures [1]. We used a standard clinical method to assess touch sensory perception, with specific applications to somatosensory impairment, allowing a simple and effective approach [30].

In accordance with our hypothesis, in those children perceiving stimuli, the MMC group perceived light touch sensation less clearly than TD children at all tested sites, whereas we found no significant differences between TD and AMC groups. The latter group even performed better than MMC-affected children in terms of the first metatarsal head, fifth metatarsal, and mid-dorsum. Similarly, in the two-point discrimination, our findings indicated that participants in the MMC group discriminated increasingly poorly on both the forefoot and heel compared to the TD and AMC groups. This also applies to vibration, which was found to be perceived for a significantly shorter time by MMC participants than by TD and AMC participants on the first metatarsal head and the medial malleolus.

However, except for the TD children, not all participants in our study group reached the lowest threshold for perceiving the stimuli during the sensory testing. Of the MMC participants, the largest monofilament during light touch pressure testing could not be identified by approximately 22% to 35%, and this occurred more frequently with increasing muscle weakness. This finding may lead to the proposal that this somatosensory tool cannot be said to be useful for a heterogeneous diagnosis, such as MMC. However, despite the loss of non-identified stimuli, light touch pressure testing in the MMC group revealed statistically altered values compared to the AMC group except on the heel, mid-shin, and mid-thigh. It may, therefore, be reasonable to argue that the incapacity to sense even the thickest size of the filaments provides an indication of sensory loss, which is of importance for the rehabilitation praxis. As suggested by Zarkou et al. [24], we also examined two-point discrimination and vibration. Similarly, as for light touch pressure, approximately 39% to 43% of the MMC participants did not perceive the largest distance of two-point discrimination stimuli. The two-point discrimination test is reported to be used to assess a patient’s ability to identify two close points on a small area of skin and to determine how precise the ability to discriminate this is. It is a measure of the inability to identify objects and can be performed together with an assessment of dermatomes for light touch; it is most informative when performed with intact cutaneous sensation [31]. Nevertheless, the indication of not being capable of sensing the largest distance of 45–50 mm on an aesthesiometer on the heel or plantar side of the forefoot, as in some children with MMC, increases the risk of not identifying objects that can damage the skin and cause pressure sores. Moreover, vibration data were lacking between approximately 30% to 60% of participants with AMC and MMC, the latter number for vibration on the medial malleolus in children with AMC. This finding, again, did not seem to influence the results, showing significantly shorter perceived vibration times in children with MMC than in both children with TD and those with AMC. In children with AMC, this may have been caused by the medial malleolus being insufficiently prominent, difficult to palpate, and even, in one participant, impossible to reach. As reported by Zarkou et al. [24], children with CP perceived vibration stimulus at the first metatarsal for a longer time period than TD participants; however, they reported values that were broadly consistent with our results for both TD and AMC children. The different results could indicate that there may be a subjective factor in the measurement of the duration of the vibration stimulus in the interaction between the investigator and patient that should be taken into account. In order to provide a clearer understanding of how the vibratory function affects motor performance, further exploration is recommended [24].

Unlike what has been reported in the literature, which has shown proprioceptive deficits in the distal form of AMC [9,10], we could not confirm a lack of proprioception in AMC patients after measuring kinesthesia in the ankle joint. Among the heterogenous group of children with AMC, normal sensation was reported in amyoplasia [7,8], which seems consistent with the findings of this study, even though the findings have to be interpreted with caution due to the low number of participants. As could be expected, participants with MMC at a lower ambulatory level and with higher lesion levels perceived ankle kinesthesia less than those with higher functions; this may point to an increased need for orthotic stabilization of the lower limb joints. Compared to the study of Zarkou et al. [24], we included children with at least five degrees of ankle ROM, while Zarkou et al. required 30 to 40 degrees of plantarflexion to be included, which could not have been a relevant demand for the children with AMC and MMC in this study.

Among the children with MMC, 52% had orthoses encompassing the ankle and knee joints, whereby only a small proportion reported skin irritation, as assessed on the examination day. This is remarkable among MMC participants, who have decreased tactile sensation, and therefore, it can be assumed that these children had properly fitted orthoses. In patients with flaccid paresis, few skin irritations may occur because skin integrity may be maintained if there is a loss of sensation, even over the entire sole of the foot [32]. This could be relevant for this MMC study group, where skin irritations were reported by only five participants. These were reported on the back, on the first toe, on the fifth toe, and, by two participants, “sometimes” on the feet. However, as reported based on a national registry study, including 180 participants, pressure injuries were common even in young children with MMC. In many cases, these were related to orthosis use, and detection at an early stage was recommended [33]. Children with disabilities may also have an altered load on their legs and feet as a result of a more pronounced muscular imbalance and deformities. These consequences can lead to an uneven distribution of load to local areas of peak pressure [32]. In cases of AMC, where foot deformities are common, no skin irritation was reported. Instead, four participants reported pain being localized to the metatarsal side and down under the foot, medially scored on the VAS as “6”; one child reported pain in their feet under load as VAS “5”, one reported pain on their frontal shins as VAS “6”, and one participant reported pain in their “left heel all around and at the ankle in the evening” as VAS “7”. One participant in the AMC1 group used an orthosis covering the trunk and did not report perceived pain; however, in the AMC group, 68% of the participants used orthoses covering the ankles and knee joints. In the five participants with MMC who reported pain, it was localized to the calf as VAS “6”, in the right hip as VAS “10”, in the left hip and knee as VAS “6” and “2”, in the lumbar back as VAS “6”, and as back pain associated with pain in both legs. This more proximally distributed pain may be due to orthopedic factors and/or alterations in the spinal cord that can occur during the growing years in persons with MMC [11]. For explaining the children’s choices in the Faces Pain Scale, these were transformed to VAS scores [28]. 

With the use of the various floor tiles, we intended to offer an experience of sensing textures that may be present in a child’s life. We paid attention to the child initially receiving visual and tactile information about the various floors, and we allowed sensing with both feet simultaneously. Participants were considered to have passed the test if they were successful in identifying the floor texture in 3/5 attempts; this was achieved by a high percentage of children among participants with AMC compared to those with MMC. For participants with MMC, any feeling of cold on the stone floor (Floor1) did not seem to have had a decisive impact on the result, as no differences were found compared to Floor2 and Floor4. Floor3, which conveyed a slight prickling sensation, was correctly identified by the least number of participants. Floor4, with a surface of hard plastic sticks, on the other hand, could also be identified by children with MMC with higher neurological loss. It turned out that not all children had ever felt floors with their feet before, which seems logical since, in many cases (most of these for children with MMC), they use orthoses on their feet and legs for support. Therefore, it is recommended that children acquire opportunities to sense materials with their feet, even if these experiences will not have large functional consequences for daily life for many of them. In future work, we will also describe foot pressure distribution with respect to posture in standing; we will examine this with 3D motion analysis, as suggested in a preliminary study of the present work [34].

In this study, we also added light touch pressure measurements of the mid-dorsum of the foot, mid-shin, and mid-thigh [23] to obtain information about the leg as well. All children with MMC and AMC were included based on the fact that they have full neurological function in their knee extensors. The finding that nearly all had normal sensations in their mid-thigh strengthens the thesis that motor and sensory function usually follow motor loss within one or two segments [15,35]. Even though MMC is defined by the presence of sensory pareses [8], there is no validated tool to examine the lack of sensation in children with MMC. In reference to motor innervation segments [36], the sensory segment levels, with respect to light touch pressure used in this study, are referred to as L5 for the first metatarsal, S1 for the fifth metatarsal, S1 for the heel, L5 for the mid-dorsum, L4/L5 for mid-shin, and L3 for the mid-thigh.

EQ-5D-Y has been found to constitute a useful tool to measure the self-reported health status of young people in an age-appropriate manner [29]. In a previous study based on Swedish populations [37], children with AMC and MMC reported similar results in most of the five dimensions. In another study [38], the answers of children with AMC regarding the “looking after myself” dimension were proposed to be attributable to upper limb involvement, which is frequently observed in this patient group [2]. This is in accordance with the present study, in which approximately 40% of children with AMC reported problems with “looking after myself”, including problems in carrying out washing or dressing. In this study, this was reported by approximately 30% of MMC participants; this may point to hand function that is less affected compared to persons with AMC. In the “performing regular activities” dimension, such as going to school or participating in hobbies, sports, playing, or activities with family or friends, about 50% of children in the MMC group reported problems compared to about 30% of children in the AMC group. However, both groups stated similar answers in regard to the “mobility dimension (walking about)”. In the MMC group, more than 50% reported problems with pain in EQ-5D-Y compared to the reported pain on examination day. These former answers may also be interpreted as referring to a more general perceived pain in everyday life, such as back pain and pain in the hip, with radiating pain in the legs. From adults with MMC who described the localization of their pain through drawings, it was shown that pain varies with respect to ambulation groups and is not tied to areas covered by orthoses [39]. The rated health on the vertical, graduated VAS between 0 (the worst) and 100 (the best health state one can imagine) cannot be said to differ between the general child population and children in the AMC and MMC groups in any of the above-mentioned studies and in the present study. The testing of the EQ-5D-Y instrument was conducted predominantly with healthy samples. Thus, the dimension “feeling worried, sad, or unhappy” addresses problems that are more common in children’s lives than impairments in mobility, self-care, or everyday activities [29]. In this study, 35% of children with AMC and 19% of children with MMC reported problems with “feeling worried, sad, or unhappy”; the possible causes of these feelings will not be further analyzed in this study.

The study has several limitations. Firstly, it can be questioned whether the small sample size meets statistical requirements. Therefore, the findings of this study need to be validated with a larger sample size containing more homogeneous subgroups. However, AMC and MMC are considered to be so-called rare diseases, and it is not easy to establish large study groups. Moreover, not all participants reached the limit for perceiving stimuli of the somatosensory test battery at all, indicating that the tests were not applicable to all children. Since the floor test was being used for the first time, we made an effort to perform it with the utmost accuracy: without vision, in a randomized order, and with five trials for each floor. However, the test needs to be validated in larger studies in the future.

The use of methods to document sensory function with as objective and valid tools as possible is recommended to ensure the documentation of sensory aspects in effective rehabilitation protocols [24]. The findings of this study confirm this recommendation, especially for children who have reduced sensation. By documenting the child’s tactile sensation, the risk of skin damage and pressure ulcers can be avoided. However, even for children who are believed to have intact sensation, it is important to document sensation carefully. This can increase the awareness and understanding, on the part of rehabilitation professionals, of the child’s possible pain experience, e.g., when using orthotics.

## 5. Conclusions

In accordance with our hypothesis, participants with MMC perceived somatosensory stimuli less clearly than TD children in all tests; this was more noticeable the higher the level of the lesion and the more that ambulation was affected. In children with AMC, there were some minor alterations regarding the TD group, but these were not significant. The AMC group performed even better than the MMC group in two-point discrimination and in vibration sensation, as well as in some light touch pressure tests, but there were no differences between the motor function groups or ambulation groups composed of AMC participants. Not all participants even reached the threshold of identifying the stimuli that were tested, which was most evident in the MMC group. This result also provides answers regarding the degree of perceived tactile sensation and is of importance for professionals in rehabilitation. In the AMC group, it should be considered that because bony prominences were difficult to reach with a vibrating tuning fork, there may be a lack of data. Pain was perceived by relatively few participants, and the perceived health status did not differ between children with AMC and MMC nor with respect to their TD peers.

## Figures and Tables

**Table 1:** Patient characteristics of children with arthrogryposis (AMC), children with myelomeningocele (MMC), and typically developing children (TD) and subgroups of diagnoses, motor function, functional ambulation, and orthosis use in AMC and MMC groups. “n” indicates number within each group.

Median [Min, Max]	AMC (n = 19)	MMC (n = 23)	TD (n = 22)	*p*-Value
Age	10.6 [7.2, 17.9]	12.4 [7.2, 17.9]	12.1 [7.1, 17.9]	0.734
Weight	29 [19.3, 60]	40 [22, 90]	47.7 [21.7, 71.3]	0.074
Height	130 [115, 173]	143 [118, 181]	155 [120, 184]	0.052
BMI	16 [13, 27]	19 [12, 33]	19 [15, 23]	0.095
Sex (female/male)	8/11	9/14	15/7	0.107
Sub-diagnosis (n)	Amyoplasia: 5 Larsen syndrome: 1 Pterygium: 1 Freeman–Sheldon: 1 Sheldon–Hall distal 2B: 1 Distal form type 5D: 1 AMC myopathy: 2 Unclear etiology: 7	MMC: 20 Lipo-MMC: 3	-	-
Motor function (n)	AMC1: 4 AMC2: 9 AMC3: 6	MFC I: 7 MFC II: 7 MFC III: 8 MFC IV: 1	-	-
Functional ambulation	Ca:13 Ha: 2 N-f: 3 N-a: 1	Ca:12 Ha: 3 N-f: 7 N-a: 1	-	-
Orthoses (n/%)	19/100	19/83	-	-
Bilateral/unilateral	18/1	18/1		
limbs (n)				
Insole	10	10		
AFO	13	10		
KAFO-F	4	10		
KAFO-L	8	5		
THKAFO	1 (both legs)	1 (both legs)		

MFC = muscle function class, Ca = community ambulation, Ha = household ambulation, N-f = non-functional ambulation, N-a = non-ambulation, AFO = ankle–foot orthosis, KAFO-F = knee–ankle–foot orthosis with free-articulated knee joint, KAFO-L = knee–ankle–foot orthosis with lockable knee joint, THKAFO = trunk–hip–knee–ankle–foot orthosis.

**Table 2:** Light touch pressure, two-point discrimination, vibration, and kinesthesia in typically developing children (TD) and in children with arthrogryposis (AMC) and myelomeningocele (MMC). “n” indicates number of children who perceived it; * indicates *p* < 0.05.

Median [Min, Max]		TD (n = 22)		AMC (n = 19)		MMC (n = 23)	*p*-Value
Light touch pressure (Newton)	n		n		n		
First metatarsal	22	3.61 [2.83, 4.81]	19	3.61 [2.83, 6.65]	16	4.34 [2.83, 6.65]	0.014 *
Fifth metatarsal	22	3.61 [2.83, 4.81]	19	3.61 [2.83, 6.65]	15	4.34 [2.83, 6.65]	0.038 *
Heel	22	3.61 [2.83, 5.61]	18	3.61 [2.83, 6.65]	15	4.56 [2.83, 6.65]	0.028 *
Mid-dorsum	22	3.02 [2.83, 4.09]	19	2.83 [2.83, 6.65]	16	3.65 [2.83, 6.65]	0.004 *
Mid-shin	22	2.83 [2.83, 3.69]	19	2.83 [2.83, 6.65]	18	3.41 [2.83, 6.65]	0.057
Mid-thigh	22	2.83 [2.83, 3.69]	18	2.83 [2.83, 4.74]	22	2.83 [2.83, 6.65]	0.741
Two-point discrimination (mm)							
Plantar forefoot	22	15 [10, 17.5]	19	15 [10, 25]	13	22.5 [10, 50]	0.007 *
Heel	22	15 [10, 20]	19	15 [10, 40]	14	35 [10, 50]	<0.001 *
Vibration (seconds)							
First metatarsal	21	14.8 [6.7, 19.5]	11	18.5 [10.7, 21.9]	11	8.6 [3.9, 18,8]	<0.001 *
Med malleolus	21	11.9 [6.1, 17.4]	7	13.8 [9.4, 16.6]	16	8.1 [4, 18.4]	0.033 *
Kinesthesia ankle joint (score)	22	10 [10, 10]	18	10 [9, 10]	22	10 [6, 10]	0.085

**Table 3:** Percentage (number) of 58 children who were typically developing children (TD), children with arthrogryposis (AMC), and children with myelomeningocele (MMC) reporting “no” problems, “some” problems, or “a lot of problems” in EQ-5D-Y dimensions and the percentage of 57 children in the TD, AMC, and MMC groups reporting the VAS median value. “n” indicates number of children.

EQ-5D-Y Dimension	TD (n = 20)	AMC (n = 17)	MMC (n = 21)
	% (n)	% (n)	% (n)
Mobility (walking about)			
No problems	100 (20)	47.1 (8)	42.9 (9)
Some problems	0 (0)	47.1 (8)	38.1 (8)
A lot of problems	0 (0)	5.9 (1)	19.0 (4)
Looking after myself			
No problems	100 (20)	58.8 (10)	71.4 (15)
Some problems	0 (0)	35.3 (6)	14.3 (3)
A lot of problems	0 (0)	5.9 (1)	14.3 (3)
Performing regular activities			
No problems	90 (18)	70.6 (12)	57.1 (12)
Some problems	10 (2)	23.5 (4)	42.9 (9)
A lot of problems	0 (0)	5.9 (1)	0 (0))
Experiencing pain or discomfort			
No problems	95 (19)	64.7 (11)	47.6 (10)
Some problems	5 (1)	29.4 (5)	42.9 (9)
A lot of problems	0 (0)	5.9 (1)	9.5 (2)
Feeling worried, sad, or unhappy			
No problems	100 (20)	64.7 (11)	81 (17)
Some problems	0 (0)	35.3 (6)	19 (4)
A lot of problems	0 (0)	0 (0))	0 (0)
VAS median value (min, max)	95 (55, 100)	85 (45, 100)	88 (43, 100)

There was no significant difference among the TD, AMC, and MMC groups, as reported based on the EQ-5D-Y VAS (*p* = 0.587).

## Data Availability

The datasets generated and analyzed during the current study are available from the corresponding author upon reasonable request due to legal or ethical reasons.

## Abbreviations

TD, typically developing children; AMC, arthrogryposis multiplex congenita; MMC, myelomeningocele; MFC, muscle function class; AMC1, arthrogryposis class 1; AMC2, arthrogryposis class 2; AMC3, arthrogryposis class 3; Ca, community ambulation; Ha, household ambulation; N-f, non-functional ambulation; N-a, non ambulation; FO, foot orthosis; AFO, ankle–foot orthosis; KAFO-F, knee–ankle–foot orthosis, free articulated; KAFO-L, knee–ankle–foot orthosis, locked; THKAFO, trunk–hip–knee–ankle–foot orthosis.
